# Current and Emerging Strategies for Prediction and Diagnosis of Prelabour Rupture of the Membranes: A Narrative Review

**DOI:** 10.21315/mjms2021.28.3.2

**Published:** 2021-06-30

**Authors:** Saadia Ghafoor

**Affiliations:** Kakshal Hospital, Kakshal, Peshawar, Khyber Pakhtunkhwa, Pakistan

**Keywords:** prelabour rupture of membranes, PROM, preterm prelabour rupture of membranes, Fern test, chorioamnionitis, biochemical markers, biomarkers, foetal fibronectin, alpha-fetoprotein, placental alpha microglobulin-1, insulin-like growth factor binding protein-1, monoclonal/polyclonal antibody immunoassay tests, amniotic fluid interleukin-8, IL-8, placental protein 14

## Abstract

Prelabour rupture of membranes (PROM) refers to the disruption of foetal membranes before the onset of labour, resulting in the leakage of amniotic fluid. PROM complicates 3% and 8% of preterm and term pregnancies, respectively. Accurate and timely diagnosis is crucial for effective management to prevent adverse maternal- and foetal-outcomes. The diagnosis of equivocal PROM cases with traditional methods often becomes challenging in current obstetrics practice; therefore, various novel biochemical markers have emerged as promising diagnostic tools. This narrative review is sought to review the published data to understand the current and emerging trends in diagnostic modalities in term and preterm pregnancies complicated with PROM and the potential role of various markers for predicting preterm PROM (pPROM) and chorioamnionitis in women with pPROM.

## Introduction

Prelabour rupture of the membranes (PROM) refers to the leaking of amniotic fluid before labour onset, caused by the breakage of foetal membranes. PROM may occur at term or earlier (1, 2). It affects 5% to 10% of all pregnancies, 8% of term pregnancies and 3% of preterm pregnancies (3, 4). PROM at term is associated with adverse maternal and perinatal sequelae such as placental abruption, cord compression, cord prolapse, risk of cesarean birth, and maternal and neonatal infection (3, 5–9).

Preterm PROM (pPROM) is associated with foetal- and maternal-morbidity and mortality including umbilical cord compression and prolapse (10), oligohydramnios, placental abruption (11–13), necrotising enterocolitis, respiratory distress syndrome, foetal death (1, 14), maternal intra-amniotic and postpartum infection risks (2, 4, 15–17). Chorioamnionitis is associated with neurodevelopment handicap in preterm infants, early-onset sepsis and severe intraventricular haemorrhage (18–20).

The latent period is the interval between PROM and spontaneous labour onset and is inversely correlated with gestational age (1, 21). Most women with PROM at term experience spontaneous labour, with an incidence of 70% within 24 h, 85% within 48 h, and over 90% within 72 h of ruptured membranes (22, 23). There is a risk of developing an intra-amniotic infection (chorioamnionitis) in 6%–10% of pregnant women with PROM at term, which increases many folds with prolonged rupture of membranes (2–4, 24, 25). Prolonged rupture of membranes refers to PROM persisting for more than 24 h and is associated with an increased risk of chorioamnionitis and postpartum endometritis (7, 26–29). The neonatal infection risk is raised by 2.25 times if the rupture of membranes is prolonged for 24 h–48 h or more in a term pregnancy (24).

The etiology of PROM is thought to be multifactorial (30). However, the underlying pathophysiologic mechanisms are not well-understood (8, 31, 32). PROM at term, caused by the weakening of the chorioamniotic membranes, can further be aggravated by forceful uterine contractions during labour (1). Microbial invasion of the amniotic cavity and increased placental inflammation can lead to pPROM in around 20%–50% of pregnant women (33–35). The possible role of a genetic predisposition has also been proposed in pPROM (36–38). Feng et al. (39) explained the role of prothrombin production in pPROM caused by urea plasma parvum-induced rupture of amniotic membranes.

The presence of certain biochemical processes, such as collagen disruption within the extracellular matrix of chorioamniotic membranes and cellular changes such as programmed cell death in foetal membranes, has been suggested as the pathophysiological mechanisms in prelabour rupture of membranes (31, 32, 38, 40). Mediators, including prostaglandins, cytokines and hormones, may contribute to its role in disrupting the extracellular matrix of chorioamniotic membranes through certain enzymes, as postulated by the scientific data (31, 38, 41). Also, the biophysical stresses may facilitate the rupture of foetal membranes in the presence of these concurrent biochemical changes (31, 32). Inflammatory environment, including the infiltration of leukocytes and upregulation of proinflammatory chemokines in the choriodecidual interface, has been emphasised in the pathophysiology of PROM (41–43). Proinflammatory cytokines that may play a significant role in pPROM include interleukin-6, interleukin-8 and tumour necrosis factor-alpha (31, 44). These etiopathogenesis findings have led to the dynamic evolution and development of several biomarkers to predict and monitor intra-amniotic inflammation in women with pPROM.

There is a growing concern for rising trends in adverse outcomes associated with pPROM, despite the improvements in antenatal care delivery (45). PROM with the doubtful presentation may cause pregnant women to visit emergent care facilities unnecessarily (46). Therefore, the availability of point-of-care testing is essential for timely diagnosis and effective management of pregnant women with PROM (26). Lack of ancillary screening tests in preterm birth prediction is the major challenge in reducing the incidence of preterm delivery associated with pPROM (47). Until recently, there have been no significant changes in obstetric practice for diagnosing PROM for the last many years (48).

This narrative review summarises the novel concepts in understanding the current and emerging trends in diagnostic modalities in term and preterm pregnancies complicated with PROM. The potential role of various markers for predicting chorioamnionitis in women with pPROM has been discussed.

## Methods

This narrative review was performed through a literature search published between 1982 and 2020 using the Cochrane library and electronic database, including PubMed and Google Scholar, through Google search engine. Following keywords were used for this narrative review: prelabour rupture of membranes (PROM), preterm prelabour rupture of membranes (pPROM), Fern test, chorioamnionitis, biochemical markers, biomarkers, foetal fibronectin (fFN), alpha-fetoprotein (AFP), placental alpha microglobulin-1 (PAMG-1), insulin-like growth factor binding protein-1 (IGFBP-1), monoclonal/ polyclonal antibody immunoassay tests, amniotic fluid interleukin-8 (IL-8) and placental protein 14 (PP14). Also, combinations of the following search terms were used to retrieve information regarding the particular topics related to PROM at preterm and term pregnancy through Boolean search strategy: ‘biomarkers’ and ‘PROM’; ‘biomarkers’ and ‘preterm PROM’; ‘biochemical markers’ and ‘PROM’; ‘biochemical markers’ and ‘preterm PROM’ ‘predictive markers’ and ‘PROM’; ‘predictive markers’ and ‘preterm PROM’; predictive markers’ and ‘chorioamnionitis in preterm PROM’; ‘diagnosis’ and ‘PROM’; ‘diagnosis’ and ‘preterm PROM’. Relevant reference lists of retrieved publications were also reviewed to expand the search. A total of 106 scientific publications, including research articles published from 1982 to 2020, were selected for this narrative review using the aforementioned methodology.

## Discussion

### Current and Potential Diagnostic Strategies for PROM at Term and Preterm

Several techniques have been developed for evaluating PROM apart from the clinical examination in the last many decades. These methods include the nitrazine test, the Fern test, the test for determining fFN in cervicovaginal secretions, intra-amniotic dye injection and various rapid immunoassay tests for detecting protein marker present in the vaginal fluid (49). However, the diagnosis of PROM becomes a challenge in the presence of a slow or intermittent amniotic fluid leak, excessive blood in cervical-vaginal fluid or in the absence of classic presentation such as sudden painless ‘gush of fluid’ leaking out of the vagina (49, 50). The rupture of foetal membranes is not grossly apparent in approximately 20%–25% of pregnant women; therefore, the research for identifying an ideal diagnostic test is imperative (51). An ideal test should be non-invasive, expeditious, accurate, cost-effective, conveniently applicable and readily available.

Robust and effective intervention to manage PROM can be possible with a timely and accurate diagnosis to achieve an optimal perinatal and maternal outcome (52, 53). An incorrect diagnosis may lead to unnecessary obstetrics intervention and related effects such as iatrogenic preterm birth (54). Equivocal PROM cases may cause anxiety and inconvenience due to potential unnecessary emergent care-related hospital visits and extensive evaluation and associated healthcare costs in pregnant women. Such costs could have been avoided with the availability of at-home accurate and rapid testing to guide pregnant women in seeking timely obstetrics care when required.

A sterile speculum examination can help to identify the leaking fetal membranes by inspecting the pool of leaked amniotic fluid from cervical Os or in the vaginal vault (53, 55). However, this examination is a subjective method to diagnose PROM; therefore, it may result in an inadequate diagnostic performance (52, 56). The digital cervical examination is avoided given the risk of introducing infection; however, it can be performed in patients presenting with active labour or having an imminent delivery (57, 58). Oligohydramnios using ultrasound imaging may aid in pinpointing PROM in a pregnant woman with a clinical diagnosis of PROM (59, 60).

The conventional diagnostic approaches include the ‘Nitrazine test’ and the ‘Fern test’. Nitrazine test is a conventional test that has been used since 1938 for evaluating PROM (60, 61). This approach utilises pH testing of leaked amniotic fluid, but it may lead to equivocal results particularly if the leakage of amniotic fluid occurred after an hour or more. Fern test is a microscopic method that was first described in 1946 for diagnosing PROM (60). It involves inspecting the amniotic fluid showing arborised crystals in ‘palm leaf-pattern’ using a microscope (60). The presence of blood, cervical mucus, seminal fluid or an antiseptic solution in the amniotic fluid influences the Fern test’s diagnostic accuracy (60, 62).

fFN, a family of ubiquitous plasma proteins, may indicate the extracellular matrix’s degradation in the second and third trimesters of gestation when found in the cervicovaginal secretions (63). This test is known for being highly sensitive but with low specificity in PROM diagnosis (64). It is considered quite helpful in predicting preterm birth in the gestation from 24 to 30 weeks (65). A prospective comparative study performed on pregnant women with > 34 and < 37 gestational weeks found foetal fibronectin test having 91.8% accuracy, 94.5% sensitivity and 89.1% specificity in diagnosing PROM and compared it with Fern test (81.4% accuracy, 84.5% sensitivity and 78.2% specificity) and Nitrazine test (84.1% accuracy, 87.3% sensitivity and 80.9% specificity) (59). As described in this study, a simple bedside fFN test had better sensitivity and specificity than the Fern and Nitrazine tests in diagnosing PROM (59). Several caveats concerning the fFN include the risk of a false-positive result in preterm labour, pregnancy exceeding 34 weeks and after vaginal manipulation in pregnant women with intact foetal membranes (66).

Traditional approaches to diagnose ruptured foetal membranes are associated with certain limitations such as high subjectivity and low sensitivity (51). The researchers are more proactive in identifying novel biomarkers for screening and diagnosing PROM at term and preterm, particularly in equivocal or complex cases (51). Therefore, recent research work has been revolving around the utility of biomarker testing, including insulin-like growth factor binding protein-1 (IGFBP-1) and placental alpha macroglobulin 1 (PAMG-1) testing (51, 61, 67–71). The biochemical markers that detect specific proteins in the amniotic fluid may perform better compared to the conventional methods; however, such testing may give false-positive results in the presence of blood (72).

PAMG-1 is a glycoprotein produced by the placental decidual cells throughout the pregnancy. This specific glycoprotein is thought to transudate through the pores of the foetal membranes during contractions of the uterus or by inflammation-induced degradation of the extracellular matrix of fetal membranes in an infection or during labour (73, 74). Abdelazim et al. (66) compared the accuracy of fFN versus PAMG-1 for detection of PROM in pregnant women with > 34 and < 37 gestational weeks and found that a particular test based on detection of PAMG-1, had 97.3% sensitivity, 98.2% specificity, 97.7% accuracy, 98.2% positive predictive value (PPV) and 97.3% negative predictive value (NPV), when compared with fFN test that was found to have 94.5% sensitivity, 89.1% specificity, 91.8% accuracy, 89.7% PPV and 94.2% NPV. The authors of this study found this PAMG-1 testing particularly helpful in certain clinical scenarios of suspected PROM with the false-positive result of fFN (66).

Phosphorylated insulin-like growth factor binding protein-1 (phIGFBP-1) is synthesised by the placental decidual cells and is believed to be released into the cervicovaginal secretions after the tissue damage at the choriodecidual interface (75). Researchers evaluated the dipstick method for detecting the amniotic fluid in the vagina using a commercial kit with monoclonal antibodies to IGFBP-1. They found this method had a sensitivity of 95.7% and specificity of 93.1% in detecting amniotic fluid in PROM (76). In the 1990s, foetal fibronectin and phosphorylated IGFBP-1 were used to represent markers for premature rupture of membranes; however, they were later identified as biomarkers for predicting preterm birth (72).

The meta-analysis of research studies involving various biochemical markers testing in the PROM diagnosis showed no difference in terms of the performance of PAMG-1 and IGFBP-1 tests when employed in a research setting with the same clinical scenarios (67). A meta-analysis by Ramsauer et al. (77) investigated tests that were employed for diagnosing the rupture of foetal membranes by detecting IGFBP-1 and PAMG-1 in pregnancies with 25 to 37 gestational weeks. This study identified that the PAMG-1 detecting test had higher accuracy than the IGFBP-1 detecting test in pregnant women with an unknown membranes status (77). Also, several other comparative research studies found PAMG-1 superior to phIGFBP-1 for detecting amniotic fluid (72, 78, 79). Researchers also found, the test detecting PAMG-1 was significantly less likely to be influenced by the presence of blood compared with the test detecting IGFBP-1 in patients presenting with signs and symptoms of rupture of foetal membranes and vaginal bleeding (79). Moreover, their data showed better performance of the PAMG-1 compared with the IGFBP-1 detecting tests in all quality parameters that were evaluated (97.8% sensitivity, 91.5% specificity, 94.6% PPV, 96.4% NPV for PAMG-1 tests versus 91.0% sensitivity, 75.0% specificity, 83.5% PPV, 85.7% NPV for IGFBP-1 tests (79).

Recent studies have suggested combined monoclonal/polyclonal antibody immunoassay tests for identifying certain proteins in amniotic fluid (52). These non-invasive tests are deemed to detect amniotic fluid-specific proteins with accuracy, even in the presence of cervical-vaginal fluid. Initial first-generation immunoassays were meant to utilise the monoclonal antibody approach to detect IGFBP-1 and PAMG-1 (80). In recent years, the new combination monoclonal/polyclonal antibody immunoassays point-of-care tests for diagnosing rupture of membranes are developed. One such approach detects IGFBP-1 and alpha-fetoprotein (AFP), and another immunoassay test utilises the detection of placental protein 12 (PP12) and AFP (52, 80). These immunoassays are expected to provide quick and accurate results compared to conventional tests. Diagnostic performance of novel monoclonal/polyclonal immunoassays in PROM using the detection of two amniotic fluid proteins, IGFBP-1 and AFP, can be compared with that of conventional fern test. Research shows that monoclonal/polyclonal immunoassay test has 100% sensitivity, 94.8% specificity, and 95.5% accuracy, 75% PPV and 100% NPV (52). On the other hand, the conventional Fern test has 77.8% sensitivity, 79.3% specificity, and 79.1% accuracy, 36.8% PPV and 95.8% NPV (52).

A research study in the 1990’s era established the 100% diagnostic rate with bedside AFP-test kit (anti-AFP monoclonal antibody kit) in PROM or suspected PROM cases between 11th and 40th weeks of pregnancy (81). Kishida et al. (82) found an improved AFP test with 95.7% diagnostic accuracy through utilising AFP monoclonal antibody in pPROM. A systematic review by van der Ham et al. (83) assessed the accuracy of various tests in diagnosing equivocal cases of PROM and compared the AFP test with methods using pH measurement and IGFBP-1. This review found the AFP test having 100% sensitivity and specificity; however, researchers could not conclude recommending a particular test given the limited evidence on the diagnostic accuracy of tests in PROM. Singh and Bhat (84) performed a prospective study to assess the efficacy of AFP test in cervicovaginal secretions to diagnose PROM with 24 gestational weeks and found this test had 88.9% sensitivity and 98.5% specificity and 93.8% accuracy, PPV of 98.3%, and NPV of 90.1%. A qualitative immunochromatographic diagnostic test has been proposed as a quick point-of-care test that uses a monoclonal/polyclonal antibody approach to detect AFP and IGFBP-1 (85). One of the commercially available immunoassay tests that detect two amniotic fluid proteins, i.e., IGFBP-1 and AFP, performs well even in blood contamination settings (86).

Researchers have also investigated the role of vaginal washing fluid’s urea and creatinine measurements as diagnostic methods in patients with PROM and found these reliable and simple diagnostic tests for PROM (87, 88). Gezer et al. (89) also suggested measuring urea and creatinine levels in the vaginal fluid to diagnose and predict delivery interval after the membranes rupture in pPROM.

Amnio-dye test is an invasive method involving intra-amniotic dye instillation using fluorescein to evaluate the equivocal cases of pPROM as described by Ireland et al. (90). Although some researchers believed it was the standard gold test, this test is not without risks such as iatrogenic PROM, infection, placental abruption and miscarriage (60).

A new non-invasive and rapid bedside test has been recently introduced for pPROM, which determines interleukin-8 (IL-8) in amniotic fluid obtained through a device referred to as cervical amniotic fluid collector (91). Amniotic fluid’s interleukin-8 is analysed to predict and monitor the intra-amniotic inflammation in pPROM (91). Nevertheless, further research is required for identifying the robust biological markers for the diagnosis of pPROM (53, 69).

The United States Food and Drug Administration (USFDA) recommended that the tests used for diagnosing the rupture of membranes should be part of the pregnant women’s overall clinical assessment that includes physical examination and other relevant evaluation (92). In its letter to the health care providers (dated 8 August 2018), USFDA mentioned that the tests for detection of ruptured membranes should not be used without overall clinical assessment due to certain concerns about ‘misuse, overreliance and inaccurate interpretation of lab test results from rupture of membranes tests used to detect rupture of membranes in pregnant women’. It further stated, “These can lead to serious adverse events, including foetal death, infection and other health complications in pregnant women” (92).

Prompt and accurate diagnosis is the hallmark of gestational-age-specific management without adverse sequelae (92). Efforts to improve PROM’s diagnostic modalities should be put together through more scientific research in the future.

## Emerging Markers for Predicting pPROM and Chorioamnionitis in Women with pPROM

### Potential Markers for Prediction of pPROM

Prediction of pPROM relies on the identification of associated risk factors and the use of specific biomarkers. This strategy can help in reducing the incidence of preterm birth related to pPROM (72). Researchers should further evaluate the prediction testing and diagnostic methods using newly proposed biomarkers to guide their rationale and safe use in clinical practice.

Underhill et al. (93) recently described a serum panel comprising two proteoglycans, i.e. biglycan and decorin, together with serum protein sex hormone-binding globulin (SHBG) as a promising second-trimester prenatal serum screening-based biochemical model with an ability to predict pPROM in asymptomatic women. They found that the increased serum concentrations of biglycan in conjunction with decreased serum concentrations of decorin and SHBG in the second trimester were observed in asymptomatic patients who developed pPROM later on (93). However, more scientific research is required to target effective screening strategies for PROM at term and preterm.

Ryu et al. (94) evaluated the role of the maternal c-reactive protein (CRP) and oxidative stress markers in predicting women’s latent period with pPROM. They found these markers useful in their research. Tests measuring the urea and creatinine levels in the vaginal fluid have the potential to predict the delivery interval after pPROM (89). Köseoğlu et al. (95) suggested considering second-trimester maternal serum amyloid A (SAA) levels as a potential marker for predicting pPROM.

Toprak et al. (96) investigated platelet-to-lymphocyte ratio (PLR) as an inflammatory marker for diagnosing pPROM and found significantly higher PLR and neutrophil-to-lymphocyte ratio (NLR) in pPROM patients. These researchers found PLR with 57.8% sensitivity and 73.7% specificity and considered it a readily available, cost-effective and feasible marker for pPROM timely diagnosis (96). Sak et al. (97) found that increased levels of soluble vascular cell adhesion molecule-1 (sVCAM-1) and soluble intercellular adhesion molecule-1 (sICAM-1) levels in maternal serum and vaginal fluid can be used as biochemical markers to support the diagnosis of pPROM.

Wang et al. (49) evaluated the diagnostic value of potential biomarkers for diagnosing pPROM. Their study identified 540 unique proteins found in amniotic fluid. They selected 12 of these 540 unique proteins for further evaluation. Among those proteins, placental protein 14 (PP14) was observed with outstanding diagnostic accuracy for pPROM with 100 % sensitivity and 87.5% specificity (with a cut-off value of 0.008 μg/mL) (49). This study suggested considering PP14 as a novel potential biomarker for pPROM as it is unaffected by blood in the cervical-vaginal fluid. PP14 is a glycoprotein synthesised during pregnancy by the endometrium with high expression level in amniotic fluid (98). Further research can help in developing a bedside application using PP14 for the rapid diagnosis of pPROM.

### Potential Markers for Predicting Chorioamnionitis in Women with pPROM

pPROM increases the risk of chorioamnionitis (99). A study by Li et al. (100) evaluated the diagnostic value of CRP and procalcitonin (PCT) levels in maternal serum to predict the subclinical intrauterine infection in pregnant women with pPROM at < 34 gestational weeks and found them with good application potential. Moreover, they found PCT more applicable to pregnant women with pPROM between 28 to 33+6 gestational weeks (100). Likewise, Caloone et al. (101) found CRP the best maternal marker for predicting histological chorioamnionitis after pPROM. Similarly, Popowski et al. (102) demonstrated the association of CRP with clinical and histopathologic chorioamnionitis in PROM at or after 34 weeks of gestation.

Kunze et al. (103) studied the amniotic fluid interleukin-6 and tumour necrosis factor-α and identified these as good predictors for histologic funisitis and foetal inflammatory response syndrome in pPROM. Researchers considered giving preference to this non-invasive daily bedside sampling of amniotic fluid from cervicovaginal secretions to measure the cytokines rather than opting for invasive amniocentesis for this purpose. Martinez-Portilla et al. (104) suggested a non-invasive model consisting of maternal serum interleukin-6 and maternal characteristics. They found this model a good predictor of histological chorioamnionitis in women with confirmed pPROM.

Çakar et al. (105) studied maternal plasma presepsin level to determine its diagnostic and prognostic value for subclinical chorioamnionitis that complicates pPROM. Presepsin is a promising biomarker for inflammation that can be considered a useful marker for the early diagnosis and prognosis of various microbial infections (106). These researchers have established that presepsin level helps predict subclinical chorioamnionitis in pregnancies complicated by pPROM (105). They also found it useful in determining the optimal timing for delivery before the clinical signs of chorioamnionitis are ensued (105).

## Conclusion

Accurate diagnosis of prelabour rupture of membranes in term and preterm pregnancies is vital for timely gestational-age specific intervention and management. Well-timed prediction and prompt diagnosis of pPROM are of utmost importance. Such an approach can enable women to move to higher-level hospitals with neonatal intensive care for effective management. Apart from conventional diagnostic strategies for PROM, various current and potential emerging biomarkers derived from different body fluids (such as cervicovaginal fluid, blood and amniotic fluid) and immunoassays tests have been proposed by scientific data for diagnosis of PROM. However, the PROM diagnostic testing should be part of the patient’s overall clinical assessment, including the physical examination and relevant evaluation. The potential role of various predictive markers in predicting pPROM and chorioamnionitis in women with pPROM has also been evaluated through research work. Further robust research can help to fill the gaps in identifying the ideal diagnostic strategy and prediction testing for pregnancies complicated by PROM at term and preterm.

## References

[1] Medina TMHDA (2006). Preterm premature rupture of membranes: diagnosis and management. Am Fam Physician.

[2] (2020). Prelabor rupture of membranes: ACOG Practice Bulletin, Number 217. Obstet Gynecol.

[3] Ocviyanti D, Wahono WT (2018). Risk factors for neonatal sepsis in pregnant women with premature rupture of the membrane. J Pregnancy.

[4] Thomson AJ (2019). Care of women presenting with suspected preterm prelabour rupture of membranes from 24+0 weeks of gestation. BJOG.

[5] Tita ATN, Andrews WW (2010). Diagnosis and management of clinical chorioamnionitis. Clin Perinatol.

[6] Johnson CT, Farzin A, Burd I (2014). Current management and long-term outcomes following chorioamnionitis. Obstet Gynecol Clin North Am.

[7] Fahey JO (2008). Clinical management of intra-amniotic infection and chorioamnionitis: a review of the literature. J Midwifery Women’s Heal.

[8] Modena AB, Kaihura C, Fieni S (2004). Prelabour rupture of the membranes: recent evidence. Acta Biomed.

[9] The Royal Australian and New Zealand College of Obstetricians and Gynaecologists (2010). Term prelabour rupture of membranes (term PROM): RANZCOG statement (C-Obs 36).

[10] Bond DM, Middleton P, Levett KM, van der Ham DP, Crowther CA, Buchanan SL (2017). Planned early birth versus expectant management for women with preterm prelabour rupture of membranes prior to 37 weeks’ gestation for improving pregnancy outcome. Cochrane Database Syst Rev.

[11] Helmer H (2006). Continuing challenges in treating preterm labour: preterm prelabour rupture of the membranes. BJOG.

[12] Major CA, de Veciana M, Lewis DF, Morgan MA (1995). Preterm premature rupture of membranes and abruptio placentae: is there an association between these pregnancy complications?. Am J Obstet Gynecol.

[13] Ananth CV, Oyelese Y, Srinivas N, Yeo L, Vintzileos AM (2004). Preterm premature rupture of membranes, intrauterine infection, and oligohydramnios: risk factors for placental abruption. Obstet Gynecol.

[14] Waters TP, Mercer BM (2009). The management of preterm premature rupture of the membranes near the limit of fetal viability. Am J Obstet Gynecol.

[15] Mercer BM (2003). Preterm premature rupture of the membranes. Obstet Gynecol.

[16] Noor S, Nazar AF, Bashir R, Sultana R (2007). Prevalence of PPROM and its outcome. J Ayub Med Coll Abbottabad.

[17] Mackeen AD, Seibel-Seamon J, Muhammad J, Baxter JK, Berghella V (2014). Tocolytics for preterm premature rupture of membranes. Cochrane Database Syst Rev.

[18] Soraisham AS, Singhal N, McMillan DD, Sauve RS, Lee SK (2009). A multicenter study on the clinical outcome of chorioamnionitis in preterm infants. Am J Obstet Gynecol.

[19] Newton ER (2005). Preterm labor, preterm premature rupture of membranes, and chorioamnionitis. Clin Perinatol.

[20] Xing L, Wang G, Chen R, Ren J, Qian J, Huang Y (2019). Is chorioamnionitis associated with neurodevelopmental outcomes in preterm infants? a systematic review and meta-analysis following PRISMA. Med (United States).

[21] Shazly SA, Ahmed IA, Radwan AA, Abd-Elkariem AY, El-Dien NB, Ragab EY (2020). Middle-East OBGYN graduate education (MOGGE) foundation practice guidelines: prelabor rupture of membranes; practice guideline (01)-O-19. J Glob Health.

[22] Hannah ME, Ohlsson A, Farine D, Hewson SA, Hodnett ED, Myhr TL (1996). Induction of labor compared with expectant management for prelabor rupture of the membranes at term. N Engl J Med.

[23] Keirse MJ, Ottervanger HPSW (1996). Controversies: prelabor rupture of the membranes at term: the case for expectant management. J Perinat Med.

[24] Seaward PG, Hannah ME, Myhr TL, Farine D, Ohlsson A, Wang EE (1997). International multicentre term prelabor rupture of membranes study: evaluation of predictors of clinical chorioamnionitis and postpartum fever in patients with prelabor rupture of membranes at term. Am J Obstet Gynecol.

[25] Zanella P, Bogana G, Ciullo R, Zambon A, Serena A, Albertin MA (2010). Corionamnionite in Sala Parto. TT-[chorioamnionitis in the delivery room]. Minerva Pediatr.

[26] Caughey AB, Robinson JN, Norwitz ER (2008). Contemporary diagnosis and management of preterm premature rupture of membranes. Rev Obstet Gynecol.

[27] Mercer BM, Miodovnik M, Thurnau GR, Goldenberg RL, Das AF, Ramsey RD (1997). Antibiotic therapy for reduction of infant morbidity after preterm premature rupture of the membranes: a randomized controlled trial. J Am Med Assoc.

[28] Garite TJ, Freeman RK (1982). Chorioamnionitis in the preterm gestation. Obstet Gynecol.

[29] Beydoun SN, Yasin SY (1986). Premature rupture of the membranes before 28 weeks: conservative management. Am J Obstet Gynecol.

[30] Polzin WJBK (1998). The etiology of premature rupture of the membranes. Clin Obstet Gynecol.

[31] Parry Samuel SJF (1998). Premature rupture of the fetal membranes. N Engl J Med.

[32] Lei H, Furth EE, Kalluri R, Chiou T, Tilly KI, Elkon KB (1996). A program of cell death and extracellular matrix degradation is activated in the amnion before the onset of labor. J Clin Invest.

[33] Fortner KB, Grotegut CA, Ransom CE, Bentley RC, Feng L, Lan L (2014). Bacteria localization and chorion thinning among preterm premature rupture of membranes. PLoS One.

[34] Bopegamage S, Kacerovsky M, Tambor V, Musilova I, Sarmirova S, Snelders E (2013). Preterm prelabor rupture of membranes (PPROM) is not associated with presence of viral genomes in the amniotic fluid. J Clin Virol.

[35] Yan H, Zhu L, Zhang Z, Li H, Li P, Wang Y (2018). HMGB1-RAGE signaling pathway in pPROM. Taiwan J Obstet Gynecol.

[36] Makieva S, Dubicke A, Rinaldi SF, Fransson E, Ekman-Ordeberg G, Norman JE (2017). The preterm cervix reveals a transcriptomic signature in the presence of premature prelabor rupture of membranes. Am J Obstet Gynecol.

[37] Capece A, Vasieva O, Meher S, Alfirevic Z, Alfirevic A (2014). Pathway analysis of genetic factors associated with spontaneous preterm birth and pre-labor preterm rupture of membranes. PLoS One.

[38] Lannon SMR, Vanderhoeven JP, Eschenbach DA, Gravett MG, Waldorf KMA (2014). Synergy and interactions among biological pathways leading to preterm premature rupture of membranes. Reprod Sci.

[39] Feng L, Allen TK, Marinello WP, Murtha AP (2018). Infection-induced thrombin production: a potential novel mechanism for preterm premature rupture of membranes (PPROM). Am J Obstet Gynecol.

[40] Strauss JF (2013). Extracellular matrix dynamics and fetal membrane rupture. Reprod Sci.

[41] Gomez-Lopez N, Laresgoiti-Servitje E, Olson DM, Estrada-Gutiérrez G, Vadillo-Ortega F (2010). The role of chemokines in term and premature rupture of the fetal membranes: a review. Biol Reprod.

[42] Osman I, Young A, Ledingham MA, Thomson AJ, Jordan F, Greer IA (2003). Leukocyte density and pro-inflammatory cytokine expression in human fetal membranes, decidua, cervix and myometrium before and during labour at term. Mol Hum Reprod.

[43] Young A, Thomson AJ, Ledingham MA, Jordan F, Greer IA, Norman JE (2002). Immunolocalization of proinflammatory cytokines in myometrium, cervix, and fetal membranes during human parturition at term. Biol Reprod.

[44] Menon R, Richardson LS (2017). Preterm prelabor rupture of the membranes: a disease of the fetal membranes. Semin Perinatol.

[45] Goldenberg RL, Culhane JF, Iams JD, Romero R (2008). Epidemiology and causes of preterm birth. Lancet.

[46] Ferro C, Pyenson BS, Lau J, Kelkar M, Phillips N, Lu CW (2018). The prevalence and payer costs of potentially avoidable emergent care visits for suspected amniotic membrane rupture in pregnant women. Am Heal Drug Benefits.

[47] Mercer BM, Goldenberg RL, Meis PJ, Moawad AH, Shellhaas C, Das A (2000). The preterm prediction study: prediction of preterm premature rupture of membranes through clinical findings and ancillary testing. Am J Obstet Gynecol.

[48] Mariona FG, Cabero L (2012). Are we ready for a new look at the diagnosis of premature rupture of membranes?. J Matern Neonatal Med.

[49] Wang Y, Luo H, Che G, Li Y, Gao J, Yang Q (2018). Placental protein 14 as a potential biomarker for diagnosis of preterm premature rupture of membranes. Mol Med Rep.

[50] Workineh Y, Birhanu S, Kerie S, Ayalew E, Yihune M (2018). Determinants of premature rupture of membrane in Southern Ethiopia, 2017: case control study design. BMC Res Notes.

[51] El-Messidi A, Cameron A (2010). Diagnosis of premature rupture of membranes: inspiration from the past and insights for the future. J Obstet Gynaecol Canada.

[52] Rogers LC, Scott L, Block JE (2016). Accurate point-of-care detection of ruptured fetal membranes: improved diagnostic performance characteristics with a monoclonal/polyclonal immunoassay. Clin Med Insights Reprod Heal.

[53] Marcellin L, Goffinet F (2012). Are biological markers relevant for the diagnosis and the prognosis of preterm premature rupture of membranes (PPROM)?. Clin Chem Lab Med.

[54] Igbinosa I, Moore FA, Johnson C, Block JE (2017). Comparison of rapid immunoassays for rupture of fetal membranes. BMC Pregnancy Childbirth.

[55] Dayal S, Hong PL (2021). Premature rupture of membranes.

[56] Berger R, Abele H, Bahlmann F, Bedei I, Doubek K, Felderhoff-Müser U (2019). Prevention and therapy of preterm birth. Guideline of the dggg, oeggg and sggg (s2k level, AWMF registry number 015/025, February 2019) - part 2 with recommendations on the tertiary prevention of preterm birth and the management of preterm premature rupture of membranes. Geburtshilfe Frauenheilkd.

[57] Alexander JM, Mercer BM, Miodovnik M, Thurnau GR, Goldenberg RL, Das AF (2000). The impact of digital cervical examination on expectantly managed preterm rupture of membranes. Am J Obstet Gynecol.

[58] Munson LA, Graham A, Koos BJ, Valenzuela GJ (1985). Is there a need for digital examination in patients with spontaneous rupture of the membranes?. Am J Obstet Gynecol.

[59] Abdelazim IA (2013). Fetal fibronectin (quick check fFN test®) for detection of premature rupture of fetal membranes. Arch Gynecol Obstet.

[60] Eskicioglu F, Gur EB (2015). Diagnostic modalities in premature rupture of membranes. Int J Women’s Heal Reprod Sci.

[61] Erdemoglu E, Mungan T (2004). Significance of detecting insulin-like growth factor binding protein-1 in cervicovaginal secretions: comparison with nitrazine test and amniotic fluid volume assessment. Acta Obstet Gynecol Scand.

[62] Abdelmane A-S (2015). The evaluation of amnisure for the detection of premature rupture of membranes. Women’s Heal.

[63] Jun SY, Lee JY, Kim HM, Kim MJ, Cha HH, Seong WJ (2019). Evaluation of the effectiveness of foetal fibronectin as a predictor of preterm birth in symptomatic preterm labour women. BMC Pregnancy Childbirth.

[64] Eriksen NL, Parisi VM, Daoust S, Flamm B, Garite TJ, Cox SM (1992). Fetal fibronectin: a method for detecting the presence of amniotic fluid. Obstet Gynecol.

[65] Goldenberg RL, Mercer BM, Iams JD, Moawad AH, Meis PJ, Das A (1997). The preterm prediction study: patterns of cervicovaginal fetal fibronectin as predictors of spontaneous preterm delivery. national institute of child health and human development maternal-fetal medicine units network. Am J Obstet Gynecol.

[66] Abdelazim IA, Abdelrazak KM, Al-Kadi M, Yehia AH, Abdulkareem AF (2014). Fetal fibronectin (quick check fFN test) versus placental alpha microglobulin-1 (AmniSure test) for detection of premature rupture of fetal membranes. Arch Gynecol Obstet.

[67] Palacio M, Kühnert M, Berger R, Larios CL, Marcellin L (2014). Meta-analysis of studies on biochemical marker tests for the diagnosis of premature rupture of membranes: comparison of performance indexes. BMC Pregnancy Childbirth.

[68] Gaucherand P, Salle B, Sergeant P, Guibaud S, Brun J, Bizollon CA (1997). Comparative study of three vaginal markers of the premature rupture of membranes: insulin like growth factor binding protein 1 diamine-oxidase pH. Acta Obstet Gynecol Scand.

[69] Ghafoor S (2020). Review of prelabor rupture of the membranes: pathophysiologic concepts and novel therapeutic strategies. Curr Women’s Heal Rev.

[70] Doret M, Cartier R, Miribel J, Massardier J, Massoud M, Bordes A (2013). Premature preterm rupture of the membrane diagnosis in early pregnancy: PAMG-1 and IGFBP-1 detection in amniotic fluid with biochemical tests. Clin Biochem.

[71] Gallot D, Guibourdenche J, Sapin V, Goffinet F, Doret M, Langer B (2012). Which biological test to confirm rupture of membranes?. J Gynecol Obstet Biol la Reprod.

[72] Di Renzo GC (2019). Good clinical practice advice: prediction of preterm labor and preterm premature rupture of membranes. Int J Gynecol Obstet.

[73] Marie E, Ducarme G, Boivin M, Badon V, Pelerin H, Le Thuaut A (2020). The value of a vaginal sample for detecting PAMG-1 (Partosure®) in women with a threatened preterm delivery (the MAPOSURE study): protocol for a multicenter prospective study. BMC Pregnancy Childbirth.

[74] Lee SM, Romero R, Park JW, Kim SM, Park CW, Korzeniewski SJ (2012). The clinical significance of a positive Amnisure test in women with preterm labor and intact membranes. J Matern Neonatal Med.

[75] Conde-Agudelo A, Romero R (2016). Cervical phosphorylated insulin-like growth factor binding protein-1 test for the prediction of preterm birth: a systematic review and metaanalysis. Am J Obstet Gynecol.

[76] Darj E, Lyrenäs S (1998). Insulin-like growth factor binding protein-1, a quick way to detect amniotic fluid. Acta Obstet Gynecol Scand.

[77] Ramsauer B, Vidaeff AC, Hösli I, Park JS, Strauss A, Khodjaeva Z (2013). The diagnosis of rupture of fetal membranes (ROM): a meta-analysis. J Perinat Med.

[78] Chen FCK, Dudenhausen JW (2008). Comparison of two rapid strip tests based on IGFBP-1 and PAMG-1 for the detection of amniotic fluid. Am J Perinatol.

[79] Ramsauer B, Duwe W, Schlehe B, Pitts R, Wagner D, Wutkewicz K (2015). Effect of blood on ROM diagnosis accuracy of PAMG-1 and IGFBP-1 detecting rapid tests. J Perinat Med.

[80] Thomasino T, Levi C, Draper M, George Neubert A (2013). Diagnosing rupture of membranes using combination monoclonal/polyclonal immunologic protein detection. J Reprod Med.

[81] Kishida T (1994). Definite diagnosis of premature rupture of the membranes (PROM) using intra-amniotic dye injection method and development of a new kit (AFP-test kit) for PROM diagnosis. Hokkaido Igaku Zasshi.

[82] Kishida T, Yamada H, Negishi H, Sagawa T, Makinoda S, Fujimoto S (1996). Diagnosis of premature rupture of the membranes in preterm patients, using an improved AFP kit: comparison with ROM-check and/or nitrazine test. Eur J Obstet Gynecol Reprod Biol.

[83] van der Ham DP, van Melick MJ, Smits L, Nijhuis JG, Weiner CP, van Beek JH (2011). Methods for the diagnosis of rupture of the fetal membranes in equivocal cases: a systematic review. Eur J Obstet Gynecol Reprod Biol.

[84] Singh CR, Bhat RG (2014). Alpha-foetoprotein in the diagnosis of prelabour rupture of membranes. J Clin Diagn Res.

[85] McQuivey RW, Block JE (2016). ROM plus®: accurate point-of-care detection of ruptured fetal membranes. Med Devices Evid Res.

[86] Bushman ET, Theilen LH, Monson M, Hammad I, Esplin I, Esplin MS (2020). Effect of blood contamination on amniotic fluid detection in vitro using immunoassays. J Matern Neonatal Med.

[87] Kariman N, Afrakhte M, Hedayati M, Fallahian M, Alavi Majd H (2013). Diagnosis of premature rupture of membranes by assessment of urea and creatinine in vaginal washing fluid. Iran J Reprod Med.

[88] Kafali H, Öksüzler C (2007). Vaginal fluid urea and creatinine in diagnosis of premature rupture of membranes. Arch Gynecol Obstet.

[89] Gezer C, Ekin A, Golbasi C, Kocahakimoglu C, Bozkurt U, Dogan A (2017). Use of urea and creatinine levels in vaginal fluid for the diagnosis of preterm premature rupture of membranes and delivery interval after membrane rupture. J Matern Neonatal Med.

[90] Ireland KE, Rodriguez EI, Acosta OM, Ramsey PS (2017). Intra-amniotic dye alternatives for the diagnosis of preterm prelabor rupture of membranes. Obstet Gynecol.

[91] Oh KJ, Lee JH, Romero R, Park HS, Joon-Seok H, Yoon BH (2020). A new rapid bedside test to diagnose and monitor intra-amniotic inflammation in preterm PROM using transcervically collected fluid. Am J Obstet Gynecol.

[92] William M (2018). Risks associated with use of rupture of membranes tests — letter to health care providers.

[93] Underhill LA, Avalos N, Tucker R, Zhang Z, Messerlian G, Lechner B (2020). Serum decorin and biglycan as potential biomarkers to predict PPROM in early gestation. Reprod Sci.

[94] Ryu HK, Moon JH, Heo HJ, Kim JW, Kim YH (2017). Maternal c-reactive protein and oxidative stress markers as predictors of delivery latency in patients experiencing preterm premature rupture of membranes. Int J Gynecol Obstet.

[95] Köseoğlu SB, Guzel AI, Deveer R, Tokmak A, Engin-Ustun Y, Ozdas S (2014). Maternal serum amyloid A levels in pregnancies complicated with preterm prelabour rupture of membranes. Ginekol Pol.

[96] Toprak E, Bozkurt M, Dinçgez Çakmak B, Özçimen EE, Silahlı M, Ender Yumru A (2017). Platelet-to-lymphocyte ratio: a new inflammatory marker for the diagnosis of preterm premature rupture of membranes. J Turkish Ger Gynecol Assoc.

[97] Sak S, Barut M, Incebiyik A, Ağaçayak E, Kirmit A, Koyuncu I (2019). Comparison of sVCAM-1 and sICAM-1 levels in maternal serum and vaginal secretion between pregnant women with preterm prelabour ruptures of membranes and healthy pregnant women. J Matern Fetal Neonatal Med.

[98] Rachmilewitz J, Riely GJ, Huang JH, Chen A, Tykocinski ML (2001). A rheostatic mechanism for T-cell inhibition based on elevation of activation thresholds. Blood.

[99] Satar M, Turhan E, Yapicioglu H, Narli N, Ozgunen FT, Çetiner S (2008). Cord blood cytokine levels in neonates born to mothers with prolonged premature rupture of membranes and its relationship with morbidity and mortality. Eur Cytokine Netw.

[100] Li K (2016). Predictive value of procalcitonin or c-reactive protein for subclinical intrauterine infection in patients with premature rupture of membranes (PROM). J Prenat Med.

[101] Caloone J, Rabilloud M, Boutitie F, Traverse-Glehen A, Allias-Montmayeur F, Denis L (2016). Accuracy of several maternal seric markers for predicting histological chorioamnionitis after preterm premature rupture of membranes: a prospective and multicentric study. Eur J Obstet Gynecol Reprod Biol.

[102] Popowski T, Goffinet F, Maillard F, Schmitz T, Leroy S, Kayem G (2011). Maternal markers for detecting early-onset neonatal infection and chorioamnionitis in cases of premature rupture of membranes at or after 34 weeks of gestation: a two-center prospective study. BMC Pregnancy Childbirth.

[103] Kunze M, Klar M, Morfeld CA, Thorns B, Schild RL, Markfeld-Erol F (2016). Cytokines in noninvasively obtained amniotic fluid as predictors of fetal inflammatory response syndrome. Am J Obstet Gynecol.

[104] Martinez-Portilla RJ, Hawkins-Villarreal A, Alvarez-Ponce P, Chinolla-Arellano ZL, Moreno-Espinosa AL, Sandoval-Meija AL (2019). Maternal serum interleukin-6: a non-invasive predictor of histological chorioamnionitis in women with preterm-prelabor rupture of membranes. Fetal Diagn Ther.

[105] Çakar E, Çakar ŞE, Taşan HA, Karçaaltincaba D, Şenturk MB, Koç N (2016). Diagnostic and prognostic value of presepsin for subclinical chorioamnionitis in pregnancies between 23–28 week with preterm premature rupture of the membranes. Balkan Med J.

[106] Galliera E, Massaccesi L, de Vecchi E, Banfi G, Romanelli MMC (2019). Clinical application of presepsin as diagnostic biomarker of infection: overview and updates. Clin Chem Lab Med.

